# Elevation of plasma basic fibroblast growth factor after nocturnal hypoxic events in patients with obstructive sleep apnea syndrome

**DOI:** 10.1186/2193-1801-2-260

**Published:** 2013-06-13

**Authors:** Yumi Hirata, Tsukasa Nabekura, Hidekazu Maruyama, Kazutaka Aonuma, Makoto Satoh

**Affiliations:** Division of Sleep Medicine, Graduate School of Comprehensive Human Sciences, University of Tsukuba, 1-1-1 Tennodai, Tsukuba, Ibaraki, 305-8575 Japan; Division of Clinical and Experimental Hematology, Graduate School of Comprehensive Human Sciences, University of Tsukuba, 1-1-1 Tennodai, Tsukuba, Ibaraki, 305-8575 Japan; Department of Cardiovascular Medicine, Graduate School of Comprehensive Human Sciences, University of Tsukuba, 1-1-1 Tennodai, Tsukuba, Ibaraki, 305-8575 Japan; Department of Microbiology & Immunology, University of California, 513 Parnassus Avenue, San Francisco, CA 94143 USA

**Keywords:** Sleep medicine, Obstructive sleep apnea, Polysomnography, Hypoxia, Basic fibroblast growth factor

## Abstract

Obstructive sleep apnea syndrome (OSAS) is associated with recurrent nocturnal hypoxia during sleep; this hypoxia has been implicated in the pathogenesis of cardiovascular complication. However, a useful soluble factor that is sensitively correlated with OSAS severity for the diagnosis remains unidentified. We hypothesized that systemic levels of basic fibroblast growth factor (bFGF), a hypoxia-induced cytokine, were affected by nocturnal hypoxemia in OSAS patients, and we assessed whether the degree of change in the plasma bFGF concentrations before and after nocturnal hypoxia is correlated with the severity of OSAS. Thirty subjects who had suspected OSAS and had been investigated by nocturnal polysomnography (PSG) were enrolled. Plasma bFGF and vascular endothelial growth factor (VEGF) concentrations the night before PSG and the next morning were measured by sandwich enzyme-linked immunosorbent assay. Correlations between the changes in these factors and hypoxia-associated parameters for OSAS severity were analyzed. Patients with OSAS had significantly elevated levels of plasma bFGF but not VEGF and hemoglobin after rising. The degree of change in bFGF concentrations after nocturnal apnea episodes was significantly correlated with diagnostic parameters for OSAS severity. The change in plasma bFGF levels is associated with the degree of hypoxic state in OSAS patients, implying that bFGF might be a useful soluble factor for evaluating OSAS severity.

## Background

Obstructive sleep apnea syndrome (OSAS), which is a common public health issue affecting as much as 4% of the adult population, is associated with recurrent hypoxia during sleep ( Young et al. 1993 ; Strollo and Robers 1996 ). Patients with OSAS are exposed to decreased oxygen saturation by repeated episodes of apnea and hypopnea. The oxygen desaturation events result in the development or exacerbation of cardiovascular, cerebrovascular, and metabolic diseases ( Peppard et al. 2000 ; Bradley and Floras 2009 ; Nishibayashi et al. 2008 ; Vgontzas et al. 2000 ; Marin et al. 2005 ). Hypoxia and the subsequent tissue ischemia are major pathophysiological regulators of angiogenesis ( Lavie and Lavie 2009 ). Increased angiogenesis rates and the elevation of hemoglobin (Hb) levels in response to hypoxia are part of an adaptive response aimed at achieving increased delivery of oxygen and nutrients to the tissues ( Bunn and Poyton 1996 ; Bicknell and Harris 2004 ).

On a per-cell basis in hypoxic tissues, the transcriptional response of mammalian cells to hypoxia is largely mediated by hypoxia-inducible factor-1 (HIF-1) ( Wang et al. 1995 ). HIF-1 is a basic helix-loop-helix transcription factor composed of a HIF-1β and a HIF-1α, the production of which is strongly upregulated under hypoxic conditions ( Acker and Plate 2003 ). Vascular endothelial growth factor (VEGF) is a soluble factor that regulates multiple functions of endothelial cells ( Forsythe et al. 1996 ). Expression of VEGF is rapidly induced by HIF-1-mediated transcriptional activation in response to low oxygen levels *in vivo* as well as *in vitro* ( Acker and Plate 2003 ; Forsythe et al. 1996 ). Previous studies have reported that levels of VEGF in the peripheral blood are elevated in patients with OSAS ( Imagawa et al. 2001 ; Schulz et al. 2002 ; Lavie et al. 2002 ; Gozal et al. 2002 ). Basic fibroblast growth factor (bFGF) is also known to be strongly upregulated by HIF-1 in response to hypoxic conditions ( Calvani et al. 2006 ). It is recently reported that bFGF-dependent induction of HIF-1α expression results in the formation of an HIF-1α-bFGF positive feedback amplification pathway under hypoxic conditions in human umbilical vascular endothelial cells and rat cardiac microvascular endothelial cells *in vitro*, although VEGF, unlike bFGF, does not enhance HIF-1 gene expression ( Calvani et al. 2006 ; Li et al. 2002 ). The existence of an HIF-1α-bFGF autocrine loop suggests that the systemic level of bFGF, but not VEGF, is likely to be strongly correlated with the degree of hypoxic state in OSAS patients. However, thus far no report has demonstrated the relationship between the severity of OSAS and levels of bFGF in patients with the disease. Furthermore, a definitive diagnostic marker that is correlated with the severity of OSAS remains to be identified ( Arnardottir et al. 2009 ).

Here, we measured the plasma concentrations of bFGF, VEGF, and Hb in subjects at the night before sleep and in the next morning immediately after rising, and we verified the relationship between the degree of change in the plasma bFGF concentrations before and after nocturnally recurrent apnea episodes and the severity of OSAS.

## Results and discussion

### Characteristics of subjects

The characteristics and severity of OSAS in all subjects are summarized in Table 1. The mean ages of the two groups were similar, even though body mass index (BMI) significantly increased in the OSAS group. Apnea-hypopnea index (AHI), oxygen saturation with pulse oximetry (SpO_2_) < 90% (% total sleep time; TST), 4% oxygen desaturation index (ODI), median and minimal SpO_2_, as evaluation parameters of OSAS severity determined by polysomnography (PSG), significantly differed between the groups. All of these values were markedly higher or lower in the OSAS group than in the control group. The concentrations of VEGF and bFGF in plasma samples at night and the next morning (at 8 p.m. and at 6 a.m., respectively) were measured by sandwich enzyme-linked immunosorbent assay (ELISA) (Table 1). Unexpectedly, the plasma levels of VEGF and Hb did not differ between the groups, regardless of the morning and the night. However, the plasma bFGF levels in OSAS patients were significantly higher than those in control subjects only in the morning but not at night.

**Table 1:** **Summary of subjects and their evaluation parameters**

		Control	OSAS	***P*** value
Subject (*n*)		17	13	
Age (years)		44.3 ± 11.3	46.1 ± 12.1	6.79 × 10^-1^
BMI (kg/m^2^)		24.9 ± 2.92	29.9 ± 2.60	6.08 × 10^-4^
AHI		8.03 ± 6.51	50.9 ± 32.5	1.13 × 10^-5^
SpO_2_ < 90%	(% TST)	0.681 ± 1.31	17.8 ± 16.5	2.84 × 10^-4^
4% ODI		4.62 ± 4.23	37.1 ± 23.4	4.82 × 10^-6^
Median SpO_2_	(%)	96.4 ± 1.45	93.0 ± 3.08	6.21 × 10^-4^
Minimal SpO_2_	(%)	86.5 ± 6.72	67.6 ± 10.3	1.60 × 10^-6^
VEGF (pg/ml)	Night	14.8 ± 1.01	12.5 ± 2.24	1.68 × 10^-1^
	Morning	13.0 ± 1.85	10.3 ± 3.09	2.67 × 10^-1^
bFGF (pg/ml)	Night	2.01 ± 0.831	2.21 ± 0.573	7.47 × 10^-1^
	Morning	2.02 ± 0.743	3.43 ± 0.428	7.46 × 10^-3^
Hb (g/dl)	Night	15.2 ± 0.994	14.9 ± 0.953	6.10 × 10^-1^
	Morning	15.4 ± 1.06	15.6 ± 0.875	5.76 × 10^-1^

All participants were categorized into two groups by their OSAS severity (control, AHI ≤ 15; and OSAS, 15 < AHI). Concentrations of plasma VEGF, bFGF, and Hb the night before PSG and the next morning were measured (night and morning, respectively). Data are presented as mean values ± standard deviations. Statistical analysis for evaluation parameters among the categories was performed by the Student *t*-test.

*AHI* apnea-hypopnea index, *bFGF* basic fibroblast growth factor, *BMI* body mass index, *SpO*_*2*_ *< 90% (% TST)* percentage of total sleep time with oxygen saturation below 90%, *Hb* hemoglobin, *ODI* oxygen desaturation index, *SpO*_*2*_ oxygen saturation with pulse oximetry, *VEGF* vascular endothelial growth factor.

### Changes in VEGF, bFGF, and Hb after nocturnal apnea events

**Figure 1:**
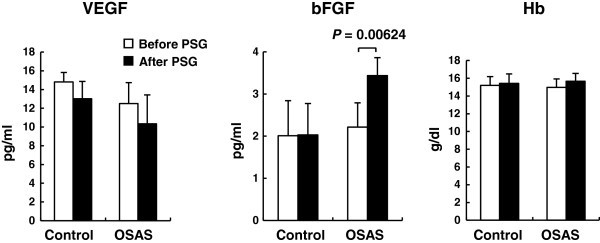
**Changes in concentrations of VEGF, bFGF, and Hb after nocturnal hypoxia.** Mean concentrations of plasma VEGF and bFGF, and Hb values the night before PSG and the next morning after PSG (before PSG, open bars; after PSG, closed bars). All subjects were categorized into two groups by their OSAS severity (control, AHI ≤ 15; and OSAS, 15 < AHI). Statistical analysis was performed by the paired *t*-test and error bars show standard deviation.

**Figure 2:**
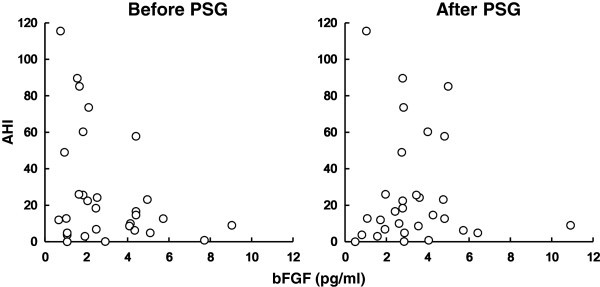
**Association between bFGF and AHI before and after nocturnal hypoxia.** Concentrations of plasma bFGF measured the night before PSG and the morning after PSG were plotted against AHI.

To assess changes in the concentrations of plasma VEGF, bFGF, and Hb after exposure to nocturnal hypoxia, we quantified these soluble factors at the night before PSG and the next morning after PSG in control and OSAS groups (Figure 1). In control subjects, the concentrations of all the factors were similar before and after sleep. In contrast, the level of plasma bFGF in the OSAS group was significantly greater after nocturnal hypoxic events than before, although there were no significant changes in VEGF or Hb (Figure 1). The elevation of plasma bFGF concentrations in OSAS patients was also confirmed in regression plots of bFGF values measured before the nigh PSG and the morning after PSG against AHI (Figure 2). These results imply that plasma levels of bFGF, unlike those of VEGF and Hb, were affected by frequent hypoxic exposure through the night in OSAS patients.

### Correlation between overnight change in VEGF and bFGF and OSAS severity

To evaluate the usefulness of the changes in plasma VEGF and bFGF levels to predict OSAS severity, we next investigated the correlation between the overnight changes in VEGF and bFGF, which were defined as ΔVEGF and ΔbFGF, respectively, and the hypoxia-associated parameters for severity of OSAS. Statistical analysis using the Pearson’s correlation coefficient revealed that ΔbFGF, but not ΔVEGF, was positively and significantly correlated with AHI, SpO_2_ < 90% (% TST) and 4% ODI, but neither age nor BMI (Table 2). ΔbFGF was also negatively and significantly correlated with median and minimal SpO_2_. These results suggest that the change in plasma bFGF might closely reflect the degree of hypoxia during sleep, i.e., the severity of OSAS.

**Table 2:** **Correlation between ΔVEGF and ΔbFGF, and OSAS severity**

	ΔVEGF (pg/ml)	ΔbFGF (pg/ml)
	rs (***P*** value)	rs (***P*** value)
Age (years)	-0.202 (1.41 × 10^-1^)	-0.242 (2.04 × 10^-1^)
BMI (kg/m^2^)	-0.145 (1.20 × 10^-1^)	0.218 (2.25 × 10^-1^)
AHI	0.00962 (2.40 × 10^-1^)	0.371 (1.17 × 10^-2^)
SpO_2_ < 90% (% TST)	0.0716 (1.77 × 10^-1^)	0.335 (1.88 × 10^-2^)
4% ODI	0.0126 (2.37 × 10^-1^)	0.354 (1.48 × 10^-2^)
Median SpO_2_ (%)	0.0907 (1.62 × 10^-1^)	-0.356 (1.69 × 10^-2^)
Minimal SpO_2_ (%)	-0.0285 (2.20 × 10^-1^)	-0.314 (1.92 × 10^-2^)

Correlations between the overnight changes (before and after PSG) in plasma VEGF and bFGF concentrations (ΔVEGF and ΔbFGF, respectively) and hypoxia-associated parameters of OSAS severity were analyzed by using the Pearson’s correlation coefficient. Coefficients of correlation are represented by rs and statistical probabilities by *P* values in parentheses.

*AHI* apnea-hypopnea index, *BMI* body mass index, *bFGF* basic fibroblast growth factor, *SpO*_*2*_ *< 90% (% TST)* percentage of total sleep time with oxygen saturation below 90%, *ODI* oxygen desaturation index, *SpO*_*2*_ oxygen saturation with pulse oximetry, *rs* Pearson’s coefficient of correlation, *VEGF* vascular endothelial growth factor.

## Discussion

Production of the cytokines VEGF and bFGF is well known to be induced by hypoxia in a HIF-1-dependent manner ( Forsythe et al. 1996 ; Calvani et al. 2006 ). We therefore hypothesized that plasma levels of these cytokines would be affected by nocturnal hypoxemia in OSAS patients. We actually indicate that levels of plasma bFGF, but not VEGF or Hb, are greater in the morning than at the night before in patients with OSAS. Notably, the change in bFGF, but not VEGF, concentration after nocturnal apnea episodes was correlated with AHI, SpO_2_ < 90% (% TST), 4% ODI, median and minimal SpO_2_. Although we could not demonstrate the role of bFGF in OSAS patients in this study, and could not completely exclude the potential cofounders that might have distributed inequally between control and study groups, these results raised the possibility that bFGF might be useful soluble factor for evaluating the severity of OSAS.

Previous studies have reported that the concentrations of various soluble factors, including VEGF, Hb, C-reactive protein, and some inflammatory cytokines in the serum or plasma, are correlated with AHI ( Imagawa et al. 2001,2004 ; Schulz et al. 2002 ; Lavie et al. 2002 ; Gozal et al. 2002 ; Ohga et al. 2003 ; Entzian et al. 1996 ; Ryan et al. 2005 ; Yokoe et al. 2003 ). In particular, VEGF has been regarded as a potential marker of OSAS ( Lavie et al. 2002 ). However, the feasibility of using VEGF has been controversial, partly because there is an age-dependent increase in systemic levels of VEGF in OSAS patients ( Valipour et al. 2004 ; Peled et al. 2007 ). Additionally, the specificity of C-reactive protein and inflammatory cytokines (e.g. tumor necrosis factor-α and interleukin-6) for OSAS seems to be low because of the high basal levels of these substances in patients with inflammatory disease, autoimmune disease, and many other diseases ( Sharma et al. 2008 ; Dinarello 1991 ; Feldmann and Maini 2001 ; Ishihara and Hirano 2002 ). This is despite the finding that comprehensive measurement of multiple cytokines has shown high levels of agreement with the AHI and may help in the diagnosis of OSAS severity ( Li et al. 2009 ).

In contrast, only a few reports have demonstrated correlations between levels of bFGF and some diseases, not including OSAS; positive correlations have been found with severe limb ischemia and specific kinds of tumors ( Rohovsky et al. 1996 ; Fujita et al. 1996 ; Clarke et al. 1998 ). We hypothesized that bFGF was strongly associated with the hypoxic condition in patients with OSAS, as suggested by the formation of a bFGF-HIF-1α amplification loop ( Calvani et al. 2006 ). In the patients, vascular endothelial cells are readily speculated to be the major source of bFGF during nocturnal hypoxia, which is a situation similar to that of upregulation of bFGF in vascular endothelial cells after the hypoxic exposure *in vitro* ( Calvani et al. 2006 ; Li et al. 2002 ). bFGF is shown to mediate a strong angiogenic activity through induction of proliferation of vascular endothelial cells as well as migration of inflammatory cells, perithelial cells, and smooth muscle cells ( Calvani et al. 2006 ; Li et al. 2002 ; Ruel and Sellke 2003 ). Therefore, bFGF is one of the feasible target molecules for therapy for ischemic heart diseases including chronic myocardial ischemia, coronary sclerosis, and myocardial infarction ( Ruel and Sellke 2003 ; Hammond and McKirnan 2001 ). In fact, bFGF is reported to have a therapeutic effect in clinical trials in coronary angiogenesis and in animal models of an ischemic heart disease and critical limb ischemia ( Nakajima et al. 2004 ; Hammond and McKirnan 2001 ; Simons et al. 2000 ). On the other hand, bFGF has a hypotensive activity ( Cuevas et al. 1991 ). Taken together, the elevation of systemic levels of bFGF after hypoxic exposure in OSAS patients is primarily supposed to have a pathophysiological role for protection of exacerbated complications of OSAS, e.g. coronary sclerosis, hypertension, ischemic heart diseases. We observed that the change of bFGF concentrations reflected the hypoxic condition in patients with OSAS during sleep periods. Moreover, our results showed the significant relevance between the change in bFGF after nocturnal hypoxic events and diagnostic parameters for OSAS severity, suggesting that it would be worth validating the clinical usefulness of bFGF. In light of our relatively small sample size, further studies of larger numbers of subjects, including OSAS patients treated by nasal continuous positive air pressure, are required to validate the reliability of bFGF as a diagnostic marker of OSAS in the clinical arena.

## Conclusions

We suggested that levels of plasma bFGF were greater in the morning than at the night before in patients with OSAS. The change in bFGF concentration after nocturnal apnea episodes was associated with the degree of hypoxic state in OSAS patients. These results raise the possibility that bFGF might be useful soluble factor for evaluating the severity of OSAS.

## Methods

### Subjects

Thirty male subjects who were suspected OSAS at the Division of Sleep Medicine participated in this study at the Tsukuba University Hospital. Male subjects with coronary artery disease, hypertension, chronic obstructive pulmonary disease, and inflammatory diseases, and female subjects were excluded in this study, because the basal levels of their plasma bFGF and VEGF might be affected by inflammation, female sex hormones, and menopausal status. All subjects with suspected OSAS were enrolled in this study and were categorized by their AHI values (control, AHI < 15; and OSAS, 15 < AHI) (Table 1). All subjects provided written informed consent after receiving a full explanation of the procedures. This study was approved by the University of Tsukuba institutional review committee and was performed in accordance with the recommendations found in the Helsinki Declaration.

BMI was calculated as the ratio of weight (kg) to height (m) squared.

### PSG

The sleep and respiratory evaluations of all participants were manually scored by specialists at the Division of Sleep Medicine, Tsukuba University Hospital. All subjects underwent a standard overnight PSG with computerized recording by the Alice-5 system (Respironics; Pittsburgh, PA). Recordings included an electroencephalogram (at positions C3/A2, C4/A1, O1/A2, and O2/A1 of the International 10–20 System), electrooculogram, submental electromyogram, left and right tibialis anterior electromyogram, thoracic-abdominal effort, oral/nasal airflow (thermistor- and pressure-based flow measurement), SpO_2_, and body position. Scoring of sleep stage and respiratory events were based on the criteria of the American Academy of Sleep Medicine ( Iber et al. 2007 ). Apnea was defined as the cessation of inspiration for no less than 10 seconds. Hypopnea was defined as a reduction in airflow by no less than 30%, with a decrease in SpO_2_ by 4% or more, for at least 10 seconds in the presence of thoracoabdominal ventilatory effort. Obstructive apnea was defined as the absence of airflow in the presence of ribcage or abdominal excursions. The AHI was calculated as the total number of apneic and hypopneic events per hour of sleep periods. All such events were counted, irrespective of the occurrence of arousal that was defined according the criteria of the American Sleep Disorders Association ( 1992 ). TST was defined as the time from the first to last recorded sleep periods, excluding wakefulness. The 4% ODI was defined as the total number of 4% desaturation of oxyhemoglobin divided by TST and was expressed as the number of events per hour. The percentage of the TST with SpO_2_ below 90% (SpO_2_ < 90% (% TST)) was also measured. An AHI of more than 15, irrespective of the presence of sleep-related symptoms (i.e. snoring, witnessed apnea, excessive daytime sleepiness), was considered as diagnostic of OSAS.

### Measurement of VEGF and bFGF in plasma

At the night before the PSG and in the next morning after the subject arose (at 8 p.m. and at 6 a.m., respectively), peripheral blood was carefully collected into tubes with buffered citrate. Plasma samples were prepared by centrifugation at 1500 rpm for 10 minutes at 20°C followed by collection of the supernatants. The supernatants were frozen at -80°C immediately after the collection in order to avoid degradation of soluble factors in them. The concentrations of plasma VEGF and bFGF were determined by sandwich ELISA with quantitative ELISA kits (R&D, Minneapolis, MN). The change in the plasma VEGF and bFGF concentrations from the night of the PSG to the next morning after rising was described as ΔVEGF and ΔbFGF, respectively.

### Statistical analysis

Statistical analysis for evaluation parameters among categories of subjects (age, BMI, AHI, SpO_2_ < 90% (% TST), 4% ODI, median and minimal SpO_2_, and plasma VEGF, bFGF, and Hb concentrations the night before PSG and the next morning) was performed by the Student *t*-test. Statistical analysis for concentrations of plasma VEGF, bFGF, and Hb before and after sleep was performed by the paired *t*-test. *P* < 0.05 is regarded as a statistically significant difference. Statistical power analysis was performed by a G*Power 3 program (Heinrich-Heine-Universität). The correlation between ΔVEGF and ΔbFGF, and AHI, SpO_2_ < 90% (% TST), 4% ODI, median and minimal SpO_2_ in each subject was evaluated with the Pearson’s correlation coefficient.

## Abbreviations

AHI: Apnea-hypopnea index
bFGF: Basic fibroblast growth factor
BMI: Body mass index
ELISA: Enzyme-linked immunosorbent assay
Hb: Hemoglobin
HIF-1: Hypoxia-inducible factor-1
ODI: Oxygen desaturation index
OSAS: Obstructive sleep apnea syndrome
PSG: Polysomnography
SpO2: Oxygen saturation with pulse oximetry
TST: Total sleep time
VEGF: Vascular endothelial growth factor
